# Unveiling neuroimmunology profile of immunological non-responders in HIV: a multimodal MRI approach

**DOI:** 10.3389/fimmu.2024.1452532

**Published:** 2024-12-13

**Authors:** Yang Zhang, Jiahao Ji, Luyao Zheng, Miaotian Cai, Guangqiang Sun, Yundong Ma, Xin Zhang, Xue Chen, Yulin Zhang, Xiao Lin, Zhen Li, Tong Zhang

**Affiliations:** ^1^ Center for Infectious Diseases, Beijing Youan Hospital, Capital Medical University, Beijing, China; ^2^ Beijing Key Laboratory of HIV/AIDS Research, Beijing, China; ^3^ Beijing Institute of Sexually Transmitted Disease Prevention and Control, Beijing, China; ^4^ Department of Respiratory and Critical Care Medicine, Beijing Youan Hospital, Capital Medical University, Beijing, China; ^5^ Beijing Key Laboratory of Mental Disorders, National Clinical Research Center for Mental Disorders and National Center for Mental Disorders, Beijing Anding Hospital, Capital Medical University, Beijing, China; ^6^ Advanced Innovation Center for Human Brain Protection, Capital Medical University, Beijing, China

**Keywords:** immunological non-responders, multimodal magnetic resonance, neuroimmune, human immunodeficiency virus, mass cytometry

## Abstract

**Background:**

People living with HIV (PLWH), especially immunological non-responders (INRs), may experience adverse neurologic events. However, the extent of neurological impairment in INRs remains uncertain. This study evaluates brain structure and function, immune dysregulation, and peripheral immunomarkers in INRs and immunological responders (IRs) among PLWH, classified according to immunological response criteria, within a clinical research setting.

**Methods:**

This study utilized multi-modal MRI to assess brain structure and function in INRs (n = 25) and IRs (n = 53). Mass cytometry and Luminex/ELISA assays were employed to analyze peripheral blood monocytes, T cell subpopulations, cytokines, chemokines, neurotrophic factors, and endocrine factors.

**Results:**

Neuroimaging findings revealed notable changes in brain structure and function in INRs, including increased fractional amplitude of low-frequency fluctuations and regional homogeneity in the left middle temporal gyrus, as well as decreased grey matter volume in the left middle temporal gyrus, left lingual gyrus, and right rolandic operculum. Furthermore, INRs exhibited significant alterations in functional connectivity in the temporal and occipital gyrus. Mass cytometry analysis demonstrated significant immune dysregulation in INRs, characterised by increased levels of senescent and activated T cells, and heightened monocyte activation. Additionally, noteworthy associations were found between neurological abnormalities and peripheral levels of immunomarkers, inflammatory cytokines, chemokines, neurotrophic factors, and endocrine factors in INRs.

**Conclusion:**

These findings underscore the associations between immune dysfunction and changes in brain structure and function, emphasizing the importance of further investigations in this field.

## 1 Introduction

People living with human immunodeficiency virus (PLWH), particularly those classified as immunological non-responders (INRs), often struggle to attain normalization of CD4^+^ T cell counts and function despite maintaining viral suppression (1, 2). The reported prevalence of INRs varies between 9% and 45% across different countries or regions (3–11). INRs are frequently accompanied by sustained immune activation and chronic inflammation (12–15). This significant immunological impairment not only contributes to HIV disease progression but also increases the risk of mortality and the development of non-AIDS-related health issues, such as adverse neurological events (16–18). Previous studies have demonstrated that low CD4^+^ T cell counts are linked to HIV-associated neurological impairment and opportunistic infections in the central nervous system (CNS) (18, 19). Consequently, INRs may be more susceptible to brain impairment.

Several studies have tried to elucidate the structural and functional brain abnormalities in PLWH. Magnetic resonance imaging (MRI) has been widely employed for exploring HIV-related neurological impairment (20). Prior research has demonstrated decreased gray matter volume (GMV) in various brain regions, including the frontal and parietal cortices, insula, cingulum, basal ganglia, thalamus, and hippocampus, in PLWH receiving antiretroviral therapy (ART) compared to uninfected individuals (21–24). HIV-associated neurological disorders are frequently associated with changes in subcortical and cortical area (25). Furthermore, INRs with poorer immune status may experience more severe neuropathy. A cross-sectional study has revealed a correlation between lower CD4^+^ T cell counts and smaller brain region volumes (26). Considering the present study’s findings, a decrease in CD4^+^ T cell counts may correlate with heightened severity of neurological impairment. Nevertheless, the research on the structural and functional brain characteristics of INRs remains limited.

The Inadequate immune reconstitution observed in PLWH can be attributed to various factors, including chronic inflammation, persistent immune activation and HIV reservoirs (27, 28). Chronic neuroinflammation associated with HIV infection has been linked to cognitive dysfunction and a range of structural and functional abnormalities in the brain of PLWH (29, 30). The CNS serves as a challenging HIV reservoir to eliminate (31), contributing to persistent viral presence that undermines immune reconstitution efforts in PLWH and exacerbates HIV-associated neurological impairment (32). This is crucial because the difficulty in eradicating HIV from the CNS directly impacts the effectiveness of antiretroviral therapies and the overall health outcomes for PLWH. However, there is a notable lack of studies focusing on the nervous system of INRs. The blood-brain barrier prevents direct entry of the virus into the brain, but its impact on the nervous system is partly mediated through the release of inflammatory factors and modulation of endocrine and neurotrophic factors (29, 33, 34). Thus, the hypothesis posited in this study is that HIV infection in INRs may impact the secretion of peripheral cytokines and chemokines due to persistent inflammation and immune activation. Additionally, this infection may also influence the concentrations of neurotrophic factors and endocrine factors related to the hypothalamic-pituitary-adrenal (HPA) axis, leading to alterations in the structure and function of the brain.

Build upon this hypothesis, we conducted a comprehensive analysis of brain structure and function, alongside peripheral immunity and endocrinology, in INRs. The primary aim of this study is to elucidate the correlation between neurological abnormalities and peripheral blood immunological markers in INRs, with the ultimate objective of enhancing our understanding of neurological impairment and informing future clinical strategies.

## 2 Materials and methods

This study has been approved by the Ethics Committee of Beijing Youan Hospital, Capital Medical University. All participants were informed about the purpose of the study, and written informed consent was obtained. The research involving human subjects was conducted in accordance with the Declaration of Helsinki of the World Medical Association revised in 2013.

### 2.1 Participants

Patients presenting with opportunistic infection, neuropsychiatric disorder, autoimmune diseases, epilepsy, traumatic brain injury, substance use disorders, or other severe diseases were excluded from the study. Only patients treated with ART and had undetectable serum viral loads were included. The clinical characteristics of the participants, including demographics, CD4^+^ T cell counts, HIV RNA load, date of HIV infection, duration of HIV infection, date of ART initiation, and duration of ART treatment, were obtained from the medical records of the AIDS research cohort at Beijing Youan Hospital. Participants with a total CD4^+^ T cell count < 500 cells/µl and/or a CD4^+^ T cell count increase < 30% from baseline at 2 years after ART initiation, while maintaining an undetectable plasma HIV RNA load, were classified into INRs. On the other hand, individuals with a total CD4^+^ T cell count > 500 cells/µl and/or a CD4^+^ T cell count increase > 30% from baseline at 2 years after ART initiation, with an undetectable plasma HIV RNA load, were classified into IRs. For each participant, plasma and peripheral blood mononuclear cell (PBMC) samples were collected and stored at −80°C and in liquid nitrogen tanks, respectively. All participants provided written informed consent, as approved by the ethics committee of Beijing Youan Hospital (2023/057).

### 2.2 Clinical assessments

Mood and sleep assessment: The Self-Rating Depression Scale (SDS) and Self-Rating Anxiety Scale (SAS) were employed to assess the levels of anxiety and depression among all participants. Mental health status was assessed using the Symptom Checklist-90 (SCL-90). The Pittsburgh Sleep Quality Index (PSQI) was employed to assess sleep quality.

Other assessments: Childhood maltreatment history was evaluated using the Childhood Trauma Questionnaire (CTQ). The assessment of alcohol craving included the administration of the Alcohol Urge Questionnaire (AUQ).

### 2.3 Neuroimaging

#### 2.3.1 Image acquisition

Resting-state functional MRI data were acquired using a 1.5 T Philips MRI Scanner at the Beijing Second Hospital. Participants were instructed to lie supine, maintain immobility, close their eyes, and remain awake. To minimize head movement, soft earplugs were employed. The resting-state functional images were obtained using the echo planar imaging (EPI) sequence, with the following parameters: repetition time/echo time (TR/TE) = 4000/25 ms, 40 slices, 64 × 64 matrix, 90°flip angle, 2.8 mm slice thickness, no gap, and 102 volumes. For 3D-T1WI: TR/TE =  2500/2.98 ms, flip angle  =  7°, matrix  =  64 × 64, field of view (FOV)  =  256 mm × 256 mm, slice thickness  =  1 mm, slices = 192, scanning time 6 min 3 s.

#### 2.3.2 Data preprocessing

Data preprocessing was conducted in Matlab R2013b by using SPM12. The first 10 images were discarded. Motion correction was performed on the functional images using the realign function. During this step, 9 participants were excluded from the analysis as their maximum displacements exceeded 2 mm in any of the x, y, or z axis or angular rotations exceeded 2 degrees after head motion correction. The standard DARTEL template in MNI space was used to drive DARTEL normalization. Then, regression of nuisance covariates was performed. After that, the functional MRI (fMRI) data were subjected to temporal band-pass filtering (0.01 – 0.08 Hz) and linear detrending. Additionally, spurious covariates were removed, such as the 6-head motion parameters obtained during the rigid body correction.

#### 2.3.3 Gray matter volume data processing

The calculation of GMV was performed using SPM12. The following steps were undertaken: (1) Individual structural images were segmented into three types of brain structures: grey matter, white matter, and cerebrospinal fluid; (2) Anatomical images were standardized to the Montreal Neurological Institute Brain Template by using the DARTEL toolkit; (3) Non-linear modulation was adopted to modulate the voxel values from density to volume; (4) Gaussian kernel Smoothing was conducted; (5) Brain GMV was quantitatively measured.

#### 2.3.4 Amplitude of low-frequency fluctuations and fractional ALFF analysis

ALFF was calculated by the Resting-State fMRI Data Analysis Toolkit (RESTplus) version 1.2. The ALFF value was calculated for each voxel, and then normalized by dividing it by the global mean value. To obtain the standardized fALFF value for each voxel, the normalized ALFF value is divided by the ALFF value over the entire frequency band.

#### 2.3.5 Regional Homogeneity analysis

ReHo analysis was conducted using RESTplus version 1.2. The Kendall coefficient of consistency (KCC) was calculated between the time series of a given voxel and the time series of its last 26 voxels. The ReHo maps were standardized by dividing the KCC value of each voxel by the mean KCC value of the whole brain. The resulting imaging data were then spatially smoothed with a Gaussian kernel with a full width at half maximum of 4 mm.

#### 2.3.6 Functional connectivity analysis

The brain regions exhibiting significant differences in ALFF/fALFF, ReHo, and GMV were selected as seeds for FC analysis. Following band-pass filtering (0.01 - 0.08 Hz) and linear trend elimination, a reference time series for each seed was obtained by averaging the resting-state fMRI (rs-fMRI) time series of voxels within each seed. Voxel-wise Pearson’s correlation coefficients were then computed between the time series within each region of interest (ROI) and the filtered time series in the remaining brain regions. To enhance normality, a Fisher’s r-to-z transformation was applied to convert the correlation coefficients in each voxel into z-scores.

### 2.4 Mass cytometry and data analysis

Heavy metal isotope-tagged monoclonal antibodies are listed in **Supplementary Table S1**. Twenty-three custom-designed antibodies were utilized to differentiate a diverse range of immune cells. These antibodies were acquired pre-conjugated from Fluidigm (South San Francisco, United States). The cell labelling procedure adhered to previously established protocols (35). The isolated PBMCs were washed and treated with Cisplatin-195Pt (Fluidigm, 201064) to eliminate diseased cells. Human TruStain FcX was employed for Fc-receptor blocking prior to antibody staining. All antibodies were utilized by the manufacturer’s instructions. Subsequently, cell samples were then washed and stained with cell surface antibodies for 30 minutes on ice. Following this, the antibody-labelled samples were washed and incubated in 125nM Cell-ID Intercalator-Ir (Fluidigm, United States) diluted in phosphate-buffered saline (PBS, Sigma-Aldrich, United States) and stored at 4°C. The samples were resuspended at a concentration of 5.5 × 10^5^ cells/mL in double-distilled water with a final concentration of 10% EQ Beads (Fluidigm, South San Francisco, United States). The samples were subsequently analyzed using the CyTOF2 mass cytometry system (Fluidigm, South San Francisco, United States).

The raw data of each sample were de-barcoded using unique mass tagged barcodes in a doublet-filtering scheme. A bead normalization method was employed to normalize the data files obtained from various batches. The data underwent meticulous gating using FlowJo 10.8.1 software to eliminate debris and dead cells. Subsequently, CD45^+^CD3^+^ cells and CD45^+^ CD3^-^ CD14^+^ monocytes were manually gated for further R language analysis. The cells were divided into different clusters using PhenoGragh clustering algorithm based on their surface marker expression levels. The t-distributed stochastic neighbor embedding (t-SNE), a visual dimensionality reduction algorithm, was applied for dimensionality reduction and visualization of the high-dimensional data. The distribution analysis of each cluster and marker expression, and differential analysis among different groups or sample types were performed in R software (version 3.6.0).

### 2.5 Cytokine and chemokine assay

A comprehensive cytokine/chemokine assay was performed on plasma samples from different groups using the EMD Millipore’s MILLIPLEX^®^ MAP Human Cytokine/Chemokine/Growth Factor Panel A MAGNETIC BEAD PANEL 96-Well Plate Assay, which utilizes the advanced Luminex^®^ xMAP^®^ technology. Capture antibody-coupled magnetic beads were utilized to selectively bind the target analytes in the plasma. The plate was then wrapped with foil and incubated with gentle agitation on a plate shaker overnight at a controlled temperature of 2–8°C. Following the incubation period, the plate was washed three times. Subsequently, the plate was incubated with detection reagents with gentle agitation on a plate shaker for 1 hour at room temperature (24 °C). Finally, the advanced Luminex^®^ 200™, HTS, FLEXMAP 3D^®^ software was employed to run the plate and analyze the resulting data.

### 2.6 Statistical analyses

The differences in ALFF/fALFF, ReHo, FC, gray matter volume between IRs and INRs were performed with a two-sample *t*-test using SPM12. The topological false discovery rate (FDR) was used for multiple comparisons correction with a significance threshold of 0.05. Pearson or Spearman correlation analyses were performed in the recovered two groups to evaluate the relationship between brain structural and functional changes (altered ALFF/fALFF, ReHo, FC and grey matter volume) and clinical variables (immunity indicators, laboratory data).

The statistical analysis was conducted using SPSS (version 27.0) and R (version 3.6.0). A chi-squared test (*χ2* test) or Fisher’s exact test was employed to compare INRs and IRs for categorical variables. Data distribution was assessed using the Shapiro-Wilk test. Non-normally distributed data were analyzed using the Mann-Whitney *U*-test to compare medians, while normally distributed data were analyzed using a two-tailed Student’s t-test to compare means. Spearman correlations were used to investigate the correlation between brain alteration and immune function. The results were performed using GraphPad Prism (version 9.5.1) for Windows and R (version 3.6.0). A *P* value < 0.05 was considered statistically significant.

## 3 Results

### 3.1 Characteristic of the participants

A total of 78 individuals with HIV infection were enrolled at Beijing Youan Hospital, Capital Medical University, from April 1, 2022, to September 31, 2022. The participants were divided into INRs (n = 25) and IRs (n = 53). Clinical characteristics are summarized in **Table 1**. The age, time of starting initial ART, duration of HIV infection, and duration of ART were matched between INRs and IRs. Initial CD4^+^ T cell counts, initial CD8^+^ T cell counts, recent CD4^+^ T cell counts, and recent CD8^+^ T cell counts and CD4/CD8 ratio were significantly lower in INRs, while initial HIV load was higher in INRs. These data provide critical insights into the neurological and immunological profiles of INRs and IRs. Additionally, we conducted clinical neuro-psychological assessments such as SAS, SDS, and CTQ scales (**Table 1**). No significant differences were observed between the INR and IR groups in these assessments.

**Table 1:** Demographic and clinical characteristics of all subject.

Demographic and clinical data	IRs (n = 53)	INRs (n = 25)	*P* value
Age, years, mean (SD)	36.53 (7.18)	39.32 (10.27)	0.169 ^a^
Sex, male (n)	53	25	
CD4^+^ T cell counts			
Initial CD4^+^ T cell counts, count/mm3, mean (SD)	447.55 (172.98)	162.08 (121.30)	<0.001 ^***,^a^^
CD4^+^ T cell counts at initiation of ART, count/mm3, median (IQR)	387.95 (333.00, 530.84)	181.12 (37.50, 260.50)	<0.001 ^***,^b^^
Recent CD4^+^ T cell counts, count/mm3, median (IQR)	702.00 (575.50, 931.00)	327.00 (215.18, 434.93)	<0.001 ^b^
CD8^+^ T cell counts			
Initial CD8^+^ T cell counts, count/mm3, median (IQR)	1066.00 (886.50, 1299.00)	952.00 (706.43, 1040.00)	0.004 ^**,^b^^
CD8^+^ T cell counts at initiation of ART, count/mm3, median (IQR)	1125.00 (883.50, 1346.91)	996.55 (714.43, 111.090)	0.018 ^*,^b^^
Recent CD8^+^ T cell counts, count/mm3, median (IQR)	980.00 (718.00, 1236.00)	702.00 (427.00, 1046.00)	0.022^*,^b^^
CD4/CD8 ratio			
Initial CD4/CD8 ratio, median (IQR)	0.41 (0.34, 0.48)	0.17 (0.06, 0.26)	<0.001 ^***,^b^^
CD4/CD8 ratio at initiation of ART, median (IQR)	0.40 (0.31, 0.48)	0.14 (0.06, 0.28)	<0.001 ^***,^b^^
CD4/CD8 ratio median (IQR)	0.81 (0.60, 0.83)	0.38 (0.26, 0.76)	<0.001 ^***,^b^^
HIV RNA load			
HIV load at initiation of ART, log10 copies/mL, mean (SD)	4.02 (0.56)	4.50 (0.89)	0.019 ^*,^a^^
Initial HIV load, log10 copies/mL, mean (SD)	4.00 (0.54)	4.39 (0.87)	0.051 ^a^
Initial ART regimen (INSTI/Non-INSTI - based regimen)	0/53	4/21	0.008 ^c^
Current ART regimen (INSTI/Non-INSTI - based regimen)	32/21	8/13	0.332 ^c^
Time to initiate ART after HIV discovery, months, median (IQR)	0.60 (0.40, 5.55)	0.70 (0.40, 1.05)	0.923 ^b^
Duration of ART, months, median (IQR)	78.30 (54.50, 101.50)	70.00 (47.95, 96.70)	0.556 ^b^
Duration of HIV infection, months, median (IQR)	86.20 (57.60, 106.80)	76.20 (48.65, 107.35)	0.626 ^b^
SAS	33.5 (28.75, 41.00)	34.00 (25.00, 41.50)	0.825 ^b^
SDS	33.50 (28.00, 45.25)	36.00 (27.00, 44.00)	0.796 ^b^
PSQI	6.00 (3.00, 9.00)	5.00 (2.00, 8.50)	0.383 ^b^
CTQ	59.00 (50.75, 62.25)	60.00 (56.50, 64.00)	0.202 ^b^
SCL-90	129.50 (106.25, 179.50)	127.00 (99.50, 177.00)	0.740 ^b^
AUQ	9.50 (8.00, 14.00)	8.00 (8.00, 13.00)	0.193 ^b^

IRs, immunological responders; INRs, immunological non-responders; BMI, body mass index; ART, antiretroviral therapy; INSTI, integrase strand transfer inhibitor; SAS, self-rating anxiety scale; SDS, self-rating depression scale; SCL-90, symptom checklist 90; PSQI, pittsburgh sleep quality index; CTQ, childhood trauma questionnaire; AUQ, alcohol urge questionnaire; ^*^: *P* < 0.05; ^**^: *P* < 0.01; ^***^: *P* < 0.001; ^a^ *t*-test; ^b^ Mann-Whitney *U* test; ^c^Chi-square and Fisher’s exact tests. IQR, Interquartile range; SD, Standard deviation. The Shapiro-Wilk test was performed to assess the normal distribution of variables. Standard deviation. A statistical significance with two-sided *P* < 0.05 was adopted.

### 3.2 Neuroimaging differences

#### 3.2.1 Gray matter volume

**Figure 1:**
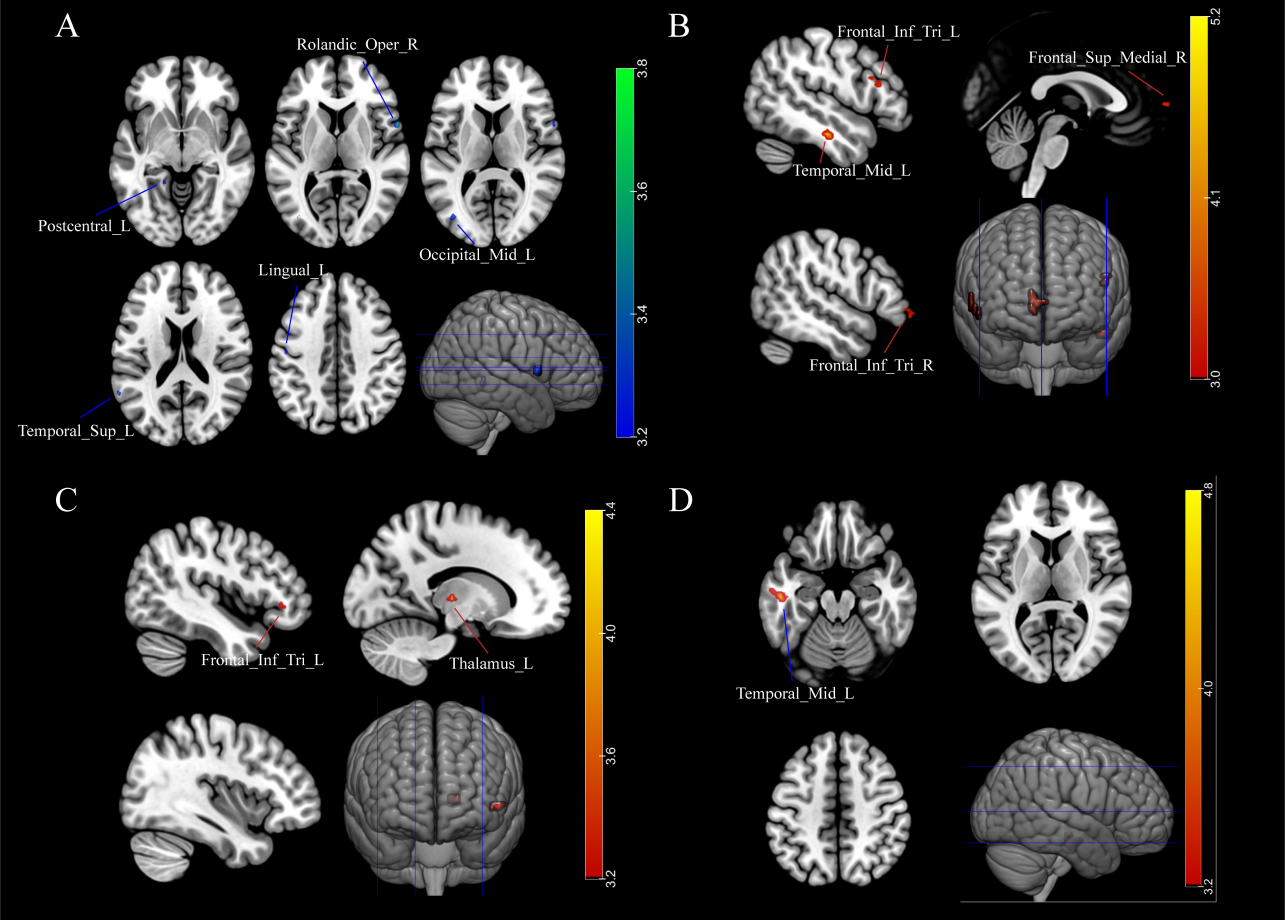
Immunological non-responders exhibit abnormal function and atrophied grey matter in their brain. **(A)** Gray matter atrophied in INRs. **(B)** Increased ALFF in INRs; **(C)** fALFF alteration in INRs; **(D)** ReHo alteration in HIV ANXs; Lingual_L: Left Lingual Gyrus; Rolandic_Oper_R: Right Rolandic Operculum; Occipital_Mid_L: Left Middle Occipital Gyrus; Temporal_Sup_L: Left Superior Temporal Gyrus; Postcentral_L: Left Postcentral Gyrus; Temporal_Mid_L: Left Middle Temporal Gyrus; Frontal_Inf_Tri_R: Right Inferior Frontal Gyrus, Triangular Part; Frontal_Sup_Medial_R: Right Superior Medial Frontal Gyrus; Frontal_Inf_Tri_L: Left Inferior Frontal Gyrus, Triangular Part; Thalamus_L: Left Thalamus. Red regions show hyperactivity, and blue regions show hypoactivity. A two-tailed two-sample t-test calculated the difference.

Compared to the IRs group, the INRs group has significantly decreased gray matter in the left lingual gyrus, right rolandic operculum (ROL), left MTG, and left postcentral gyrus (**Figure 1A**; **Supplementary Table S3**).

#### 3.2.2 Amplitude of low-frequency fluctuations and Regional Homogeneity

In this study, alterations in ALFF were observed in patients with poor immune reconstitution compared to those with immune reconstitution. To explore these differences, a two-sample t-test was performed. The results showed increased ALFF in brain regions including the left middle temporal gyrus (MTG), bilateral inferior frontal gyrus, triangular part (IFGtriang), right superior frontal gyrus, and medial (SFGmed) (**Figure 1B**). Additionally, the analysis demonstrated significantly increased fractional ALFF (fALFF) in the left IFGtriang and the left thalamus in INRs compared to IRs (**Figure 1C**). Our results indicated a significantly increased ReHo in the left MTG of the INRs group when compared to the IRs group (**Figure 1D**).

#### 3.2.3 Functional connectivity

**Figure 2:**
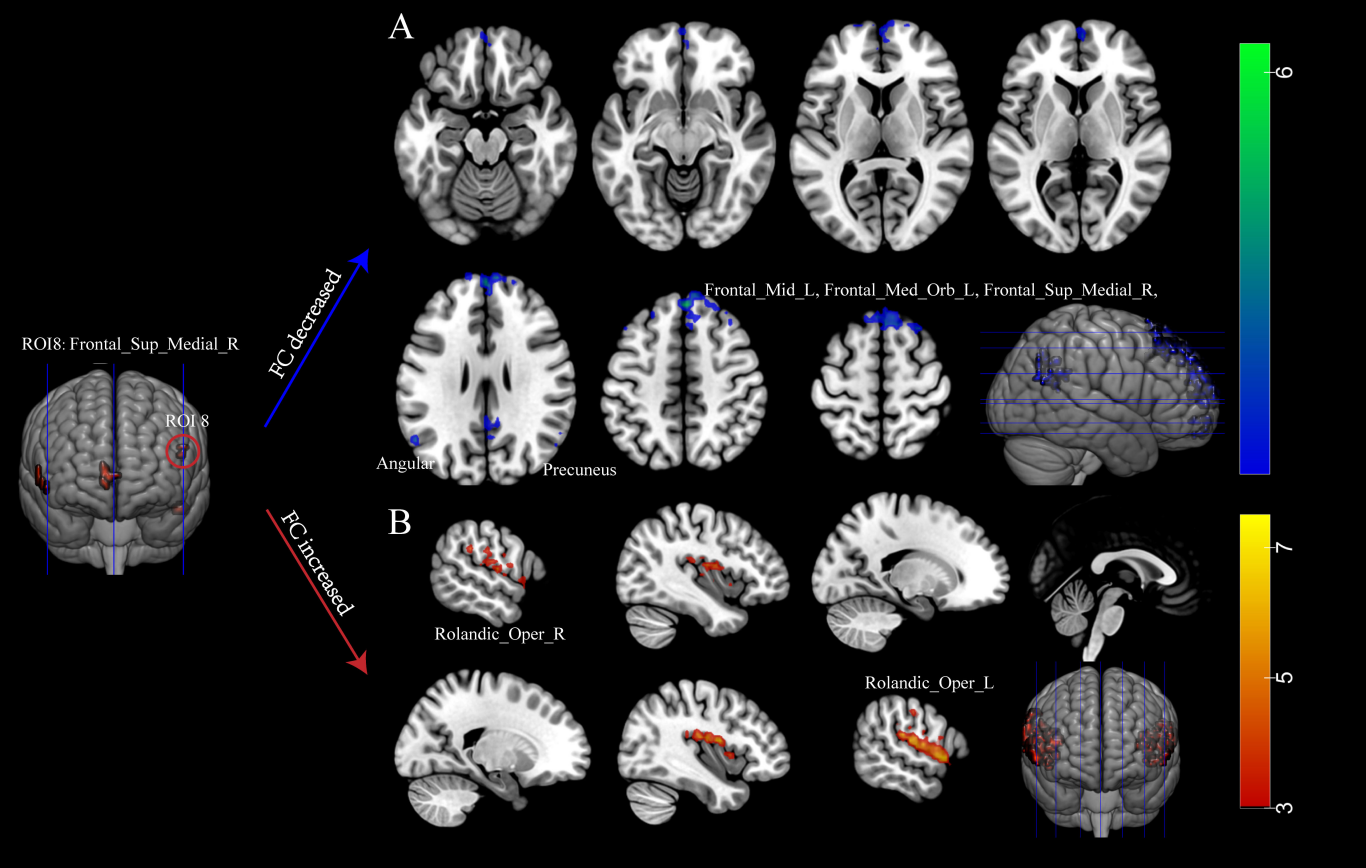
Immunological non-responders exhibit abnormal function connectivity. **(A)** Decreased FC in INRs; **(B)** Elevated FC in INRs. Red regions show hyperactivity, and blue regions show hypoactivity in FC. FC: functional connectivity; Frontal_Med_Orb_L: Left Medial Orbital Frontal Gyrus; Frontal_Sup_Medial_R: Right Superior Medial Frontal Gyrus; Precuneus_L: Left Precuneus; Angular_R: Right Angular Gyrus; Angular_L: Left Angular Gyrus; Precuneus_R: Right Precuneus; Frontal_Mid_L: Left Middle Frontal Gyrus; Rolandic_Oper_R: Right Rolandic Operculum; Rolandic_Oper_L: Left Rolandic Operculum. A two-tailed two-sample t-test calculated the difference.

In this study, nine clusters were defined as the seeds to investigate the impact of abnormal intrinsic activity on FC (**Figures 2A, B**; **Supplementary Table S4**). The defined brain regions were shown in **Supplementary Table S3**. We found notable differences in FC were observed between the right SFGmed (ROI8) and other brain regions when comparing the INRs group and the IRs group. Significant reductions in FC were detected between ROI8 and (1) left SFGmed, (2) right SFGmed, (3) left precuneus, (4) right angular, (5) left angular, (6) right precuneus, (7) left middle frontal gyrus (MFG). Conversely, a significant increase was detected between ROI8 and bilateral ROL.

### 3.3 Differences in peripheral immunity between INRs and IRs

**Figure 3:**
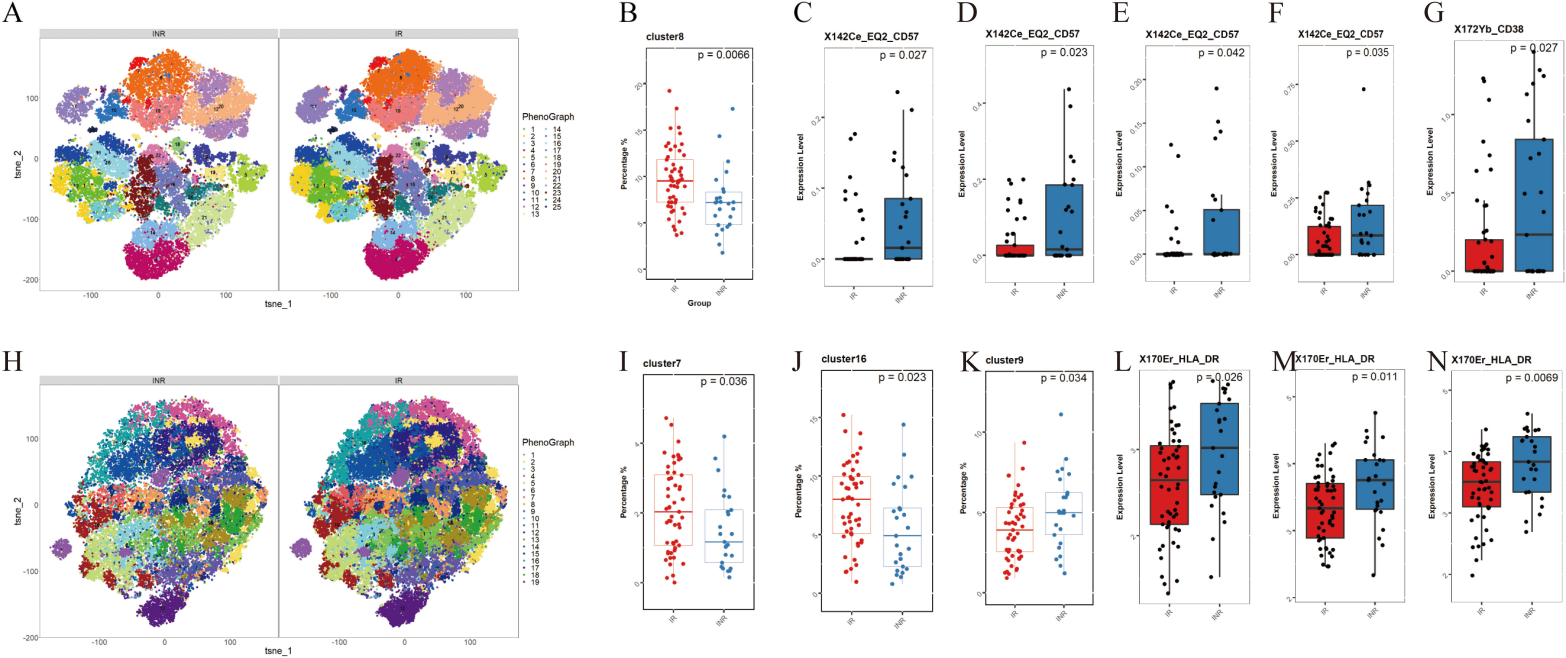
Differences in monocyte and T cell subsets between INRs and IRs. **(A)** t-SNE plots of CD3^+^ T cells in INRs and IRs, categorized into 25 clusters based on marker expression; **(B)** Decreased frequency of the C8 subpopulation (CD8^+^ central memory T cells, TCM) in INRs; **(C)** Elevated CD57 expression in CD4^+^ naive T cells (C20) in INRs; **(D, E)** Elevated CD57 expression in CD4^+^ TCM subsets (C10, C19) in INRs; **(F)** Elevated CD57 expression in CD8^+^ TCM subsets (C15) in INRs; **(G)** Elevated CD38 expression in C6 in INRs; **(H)** t-SNE plots of CD3- CD14^+^ monocytes in INRs and IRs, categorized into 19 clusters based on marker expression; **(I-K)** Decreased frequencies of classical monocytes (C7, C16) and intermediate monocytes (C9) in INRs; **(L-N)** Elevated HLA-DR expression in monocyte clusters (C7, C9, C16) in INRs, indicating heightened monocyte activation. INRs: immunological non-responders; IRs: immunological responders; t-SNE: t-distributed stochastic neighbor embedding. Differences in T cell and monocyte subpopulations were analyzed using the Mann-Whitney U-test for medians and a two-tailed Student’s t-test for means, depending on the data distribution.

We conducted mass cytometry to PBMC samples from all participants. CD45^+^ CD3^+^ T cells and CD45^+^ CD3^-^ CD14^+^ monocytes were analyzed (**Figure 3**).

#### 3.3.1 CD3^+^ T cells

CD3^+^ T cells subpopulations were categorized into 25 clusters based on the expression of markers (**Figure 3A**). **Supplementary Table S2** provides a description of the 25 clusters representing T cells subpopulations. Among these clusters, C8 (CD8^+^ central memory T cells, TCM) showed significantly lower frequencies in INRs (**Figure 3B**). When comparing the marker expression in each cluster, we observed elevated levels of CD57 expression in T cell subpopulations including CD4^+^ native T cells (TN, C20) (**Figure 3C**), CD4^+^ TCM (C10, C19) (**Figures 3D, E**), and CD8^+^ TCM (C15) (**Figure 3F**) in the INRs. Higher CD38 expression was observed in C6 (**Figure 3G**). These results indicate the presence of increased activation and senescence in specific T cell subpopulations in INRs.

#### 3.3.2 CD3^-^ CD14^+^ monocytes

Monocyte subpopulations were categorized into 19 clusters based on the expression of markers (**Figure 3H**). **Supplementary Table S2** provides a description of the 19 clusters representing monocytes subpopulation. We categorized monocytes into three subtypes: classical monocytes (CM), intermediate monocytes (IM), and nonclassical monocytes (nCM). Our findings indicate that the frequencies of CM (C7, C16) and IM (C9) were significant decreased in INRs (**Figures 3I–K**). When comparing the marker expression in each cluster, consistently elevated levels of HLA-DR were observed across clusters (C7, C9, C16) (**Figures 3L–N**). This result suggests that INRs have significantly higher levels of monocyte activation.

### 3.4 Associations between brain alterations and immunological indicators in INRs

**Figure 4:**
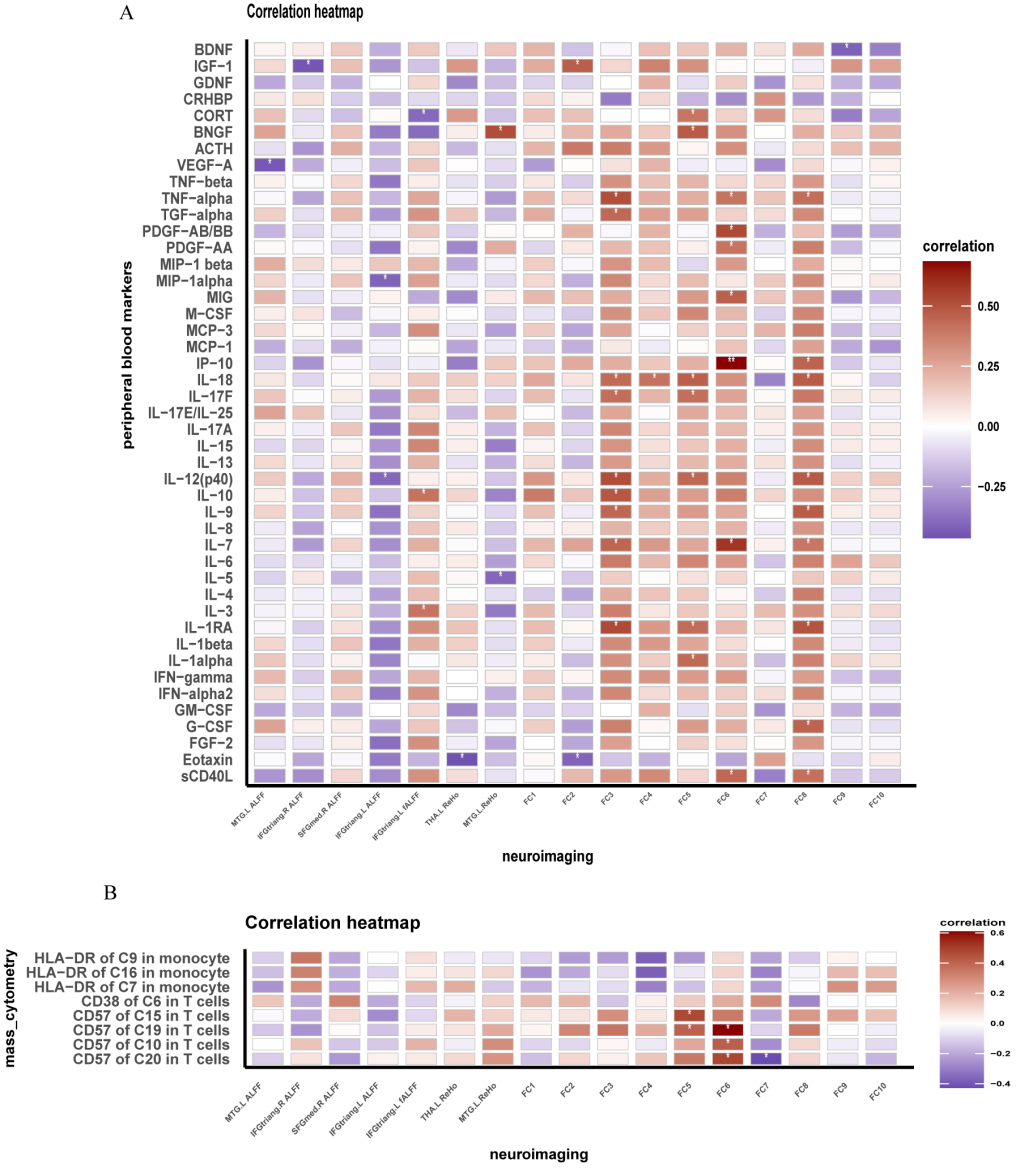
Brain functional changes in INRs correlate with peripheral inflammation and immunity. **(A)** Correlation of brain function and immune markers in INRs; **(B)** Correlation of brain function and markers expression levels of immune cells. INRs: immunological non-responders. FC1–FC9: The FC measures represent the connectivity of the Right Superior Medial Frontal Gyrus (ROI8) with the following brain regions: FC1: Frontal_Med_Orb_L; FC2: Frontal_Sup_Medial_R; FC3: Precuneus_L; FC4: Angular_R; FC5: Angular_L; FC6: Precuneus_R; FC7: Frontal_Mid_L; FC8: Rolandic_Oper_R; FC9: Rolandic_Oper_L. MTG.L: Left Middle Temporal Gyrus; IFGtriang.R: Right Inferior Frontal Gyrus, Triangular Part; IFGtriang.L: Left Inferior Frontal Gyrus, Triangular Part; THA.L: Left Thalamus; BNGF: Brain-Derived Neurotrophic Factor; MTG.L: Left Middle Temporal Gyrus; IFGtriang.R: Right Inferior Frontal Gyrus, Triangular Part; IFGtriang.L: Left Inferior Frontal Gyrus, Triangular Part; THA.L: Left Thalamus; BNGF: Brain-Derived Neurotrophic Factor. Correlation analyzed by Spearman’s correlation. ^*^: *P* < 0.05; ^**^:*P* < 0.05.

We conducted a correlation analysis in the INRs group (**Figure 4**; **Supplementary Table S5**). Our findings revealed several significant associations. In the left MTG, ALFF showed a negative correlation with VEFG. In the left IFGtriang, ALFF showed negative correlations with IL-12 (p40) and MIP-1alpha. In the left IFGtriang, fALFF demonstrated positive correlations with IL-3 and IL-10. In the left thalamus, fALFF was negatively correlated with eotaxin. FC between ROI8 and the right SFGmed exhibited a negative correlation with eotaxin. FC between ROI8 and the left precuneus showed correlations with several cytokines including IL-1RA, IL-7, IL-9, IL-10, IL-12 (p40), IL-17F, IL-18, TGF-alpha, and TNF-alpha. FC between ROI8 and the right precuneus displayed correlations with sCD40L, IL-7, IP-10, MIG, PDGF-AA, PDGF-AB/BB, and TNF-alpha. FC between ROI8 and the right angular was correlated with IL-18. FC between ROI8 and the left angular were correlated with IL-1apha, IL-1RA, IL-12 (p40), IL-17F, and IL-18. FC between ROI8 and the left MFG were correlated with sCD40L, G-CSF, IL-1RA, IL-7, IL-9, IL-12 (p40), IL-18, IP-10, and TNF-alpha (**Figure 4A**). Additionally, FC between ROI8 and the right precuneus exhibited a positive correlation with the expression of CD57 in CD4^+^ TN and CD4^+^ TCM (**Figure 4B**).

## 4 Discussion

This study provides a comprehensive examination of the neuroimmune profile in INRs by utilizing multimodal MRI techniques and assessing neuro-immunity characteristics. First, the study revealed significant alterations in volumes and activity indicators in certain brain regions in INRs. Second, INRs exhibited prominent immune dysregulation characterized by elevated levels of T cell senescence and activation, as well as heightened monocyte activation. Furthermore, we identified correlations between brain alterations and peripheral immune responses, as well as associations between altered endocrine factor levels and peripheral immune dysregulation in INRs, reflecting potential systemic dysregulation involving the immune and nervous systems. Interestingly, despite the significant differences observed in neuroimaging and immune markers between INRs and IRs, clinical neuro-psychological assessments did not show significant differences between the groups. This suggests that while neurological and immune dysfunction may be present, these changes may still be subclinical and not yet detectable through standard psychological assessments.

Consistent brain abnormalities were observed in the left MTG of INRs, including elevated ALFF and ReHo, as well as reduced GMV, compared to IRs. Previous research has also identified brain regions implicated in HIV-related neurological damage, with the temporal gyrus playing a crucial role in various cognitive functions and emotional processing. Dysfunction in the temporal gyrus, which is part of the attentional and memory networks, has been reported in PLWH (36, 37). A prior study with machine learning methodology identified brain regions specific to HIV diagnosis, revealing abnormal changes at both cortical and subcortical areas within the temporal and frontal lobes (21). Our study further observed that INRs exhibited atrophied GMV alongside heightened activity in the MTG, suggesting significant structural alterations in this region in INRs and a potentially compensatory functional response. Additionally, increased fALFF was observed in the left inferior frontal gyrus triangular part and left thalamus region, while decreased GMV was noted in the left lingual gyrus, left postcentral gyrus, and right ROL in INRs. Previous studies have indicated subcortical involvement and abnormal changes in frontal lobe structure and function in individuals infected with HIV (38, 39). GMV deficits have been documented in virally suppressed PWLH compared to uninfected controls in various regions of the brain, including the frontal and parietal cortices (22), the transitional cortex of the insula and cingulum, and subcortical structures such as the basal ganglia, thalamus, and hippocampus (21–24). These findings align with the alterations observed in immune reconstitution and suggest that inadequate immune reconstitution may worsen existing functional and structural brain abnormalities in individuals with HIV infection.

Diminished FC was observed between the right SFGmed and other brain regions in INRs. The FC analysis revealed a significant decrease in FC between ROI8 (right SFGmed) and brain regions including the right superior frontal gyrus, left MFG, bilateral precuneus gyrus, and bilateral angular gyrus, indicating disrupted functional integration within the brain networks of INRs. These regions play crucial roles in emotion regulation, cognitive function, attention, and memory (40, 41). The reduction in FC may suggest deficits in these cognitive processes and emotional regulation, potentially due to neuronal injury or inflammatory reactions related to HIV infection. Previous studies have demonstrated a significant correlation between HIV infection and changes in brain network connectivity (42–45). Changes in the right SFGmed gyrus and left MFG, regions implicated in emotion regulation and cognitive control, have been associated with mood disturbances such as depression and anxiety in individuals with HIV infection. Dysfunction in the superior frontal and medial orbital gyrus has also been documented in PLWH (46). Changes in the bilateral occipital gyrus and bilateral angular gyrus, which are known to be involved in cognitive processes such as memory, attention, and spatial cognition, have been observed in individuals with HIV infection. Dysfunction in these areas may contribute to cognitive decline, specifically deficits in attention and memory. Previous research has indicated a correlation between HIV infection and cognitive impairment, with abnormalities in the occipital gyrus and angular gyrus being implicated in this decline (47, 48). Our results suggest a potentially heightened level of brain functional impairment in INRs. Nevertheless, further research is needed to determine the extent to which this impairment may impact cognitive function and psychiatric conditions in PLWH.

Brain structural and functional alterations associated with peripheral immunity in INRs. The immunological characteristics of peripheral blood were analyzed using techniques such as mass cytometry, Luminex and ELISA in both groups. Consistent with previous research, we found higher levels of T cell activation, senescence, and higher levels of monocyte activation in INRs (49–51). Correlation analyses also provided noteworthy results, indicating potential associations between the effects of HIV infection on brain structure, function and neuro-immunity, and endocrine factors. First, brain function and peripheral inflammatory factor levels were significantly correlated. Indicators of brain activity and functional connectivity displayed correlations with inflammatory and neurotrophic factors, including IL-1, IL-3, IL-5, IL-7, IL-9, IL-10, IL-12, IP-19, eotaxin, TNF, G-CSF, sCD40L, BDNF, and IGF-1. Previous studies have linked these cytokines or chemokines to chronic inflammation in HIV infection or their involvement in neuroprotection or neuroinflammation (52–55). The correlation between these indicators may reflect responses to HIV infection-induced neuroinflammation, neurological impairment and abnormal brain network connectivity. While our study identifies significant correlations between peripheral blood markers and brain functional alterations, it is critical to acknowledge the compartmentalized nature of marker expression in the blood and CNS. These correlations should be interpreted cautiously, as peripheral markers may not fully reflect localized CNS processes due to differences in microenvironments and the restricted permeability of the blood-brain barrier (BBB). The differences in microenvironmental conditions and compartmentalized immune responses between the CNS and peripheral blood highlight the need for further mechanistic studies. Future research with integrated CSF and peripheral blood analyses is essential to validate these findings and to elucidate the causative pathways underlying these correlations. Furthermore, advanced neuroimaging techniques targeting BBB integrity could offer additional insights into the interaction between systemic immune dysregulation and CNS alterations.

Interestingly, our study revealed a significant association between brain abnormalities and endocrine factors, of which the levels of CORT were significantly correlated with changes in fALFF and FC. HIV infection disrupts biological stress pathways, such as the Hypothalamic-Pituitary-Adrenal (HPA) axis (56). Meanwhile, we identified that cortisol, a hormone involved in the HPA axis, was associated with abnormal brain function in INRs. Taken together with the previous results, we can conclude, in line with our hypothesis, that HIV in the CNS has an impact on brain structure and function in INRs, and further affects peripheral immune and inflammatory responses by modulation of hormone secretion in the HPA axis. These findings provide valuable insights for a deeper understanding of HIV-associated neuro-pathophysiologic mechanisms and suggest potential directions for future exploration of neuroprotective and therapeutic approaches.

This study demonstrates several strengths. It is the first to investigate INRs in particular, utilizing multimodal MRI to analyze brain-specific structural and functional changes. Second, the findings may provide a theoretical framework for further research on neuropsychiatric disorders in PLWH, particularly in INRs.

### 4.1 Limitations

This study has some nonnegligible limitations. First, future studies should also consider investigating biological and MRI parameters in untreated HIV participants and HIV-negative participants to provide a baseline for understanding the progression of neurological impairment without the confounding effects of ART. Second, In this study, we did not collect data on the CNS-penetration-effectiveness score of the ART regimens, which limits our ability to fully assess the impact of CNS drug penetration on neurological outcomes. The analysis of brain morphology in this study only focused on grey matter volume, which represents a limitation and emphasizes the necessity for future research to explore brain morphology in greater detail. Additionally, the precision of the 1.5T MRI machine used in this study is considered inadequate, highlighting the imperative for future studies to conduct image acquisition with higher-resolution MRI machines. The multimodal MRI and mass cytometry techniques, while resource-intensive, offer valuable insights into INRs’ neuroimmune profiles. These methods may not yet be feasible for routine use, but with further refinement, they could become more accessible. Some changes in MRI or immune parameters may be infraclinical, serving as early indicators of dysfunction before clinical symptoms emerge. Another limitation of this study is the relatively small sample size, which prevents us from developing a robust algorithm to predict neurological impairments using a small set of markers. The neurological damage observed in INRs is likely influenced by multiple factors, making it difficult to isolate specific markers at this stage. Future studies with larger cohorts will be essential to identify simplified and reliable markers for predicting brain damage in INRs, allowing for practical clinical application. While we included neuro-psychological assessment, no significant differences were observed between the INR and IR groups. This may suggest that the observed neuroimaging and immune differences may be subclinical and not yet detectable using standard psychological scales. Future studies should follow up to see whether these subclinical changes translate into clinical symptoms over time. Due to the compartmentalized expression of markers, peripheral measures may only partially represent CNS activity. Integration of cerebrospinal fluid analyses or direct CNS measures in future research would provide a more comprehensive understanding.

## 5 Conclusions

In summary, we identified significant correlations between brain structural and functional alterations and peripheral immune responses in INRs. Additionally, associations were observed between altered endocrine factor levels and peripheral immune dysregulation, reflecting potential systemic dysregulation involving the immune and nervous systems. While our findings highlight the potential impact of immune dysfunction on brain structure and function, further studies are necessary to clarify the clinical relevance and to develop strategies that effectively address both viral suppression and immune reconstitution, particularly in INRs. Our study provides a novel approach to investigating the complex interplay between immune reconstitution and neurological impairment, underscoring the importance of immune status and inflammation in this context. These findings not only contribute to our understanding of the pathophysiology of HIV-related neuropathy, but also offer valuable insights for the customization of therapeutic interventions for INRs.

## Data Availability

The raw data supporting the conclusions of this article will be made available by the authors, without undue reservation.

## References

[1] Rb-Silva R Goios A Kelly C Teixeira P Joao C Horta A. Definition of immunological nonresponse to antiretroviral therapy: A systematic review. J Acquir Immune Defic Syndr. (2019) 82:452–61. doi: 10.1097/QAI.0000000000002157 31592836

[2] Bono V Augello M Tincati C Marchetti G. Failure of cd4+ T-cell recovery upon virally-effective cart: an enduring gap in the understanding of hiv+ Immunological non-responders. New Microbiol. (2022) 45:155–72. 35920870

[3] Kaufmann GR Perrin L Pantaleo G Opravil M Furrer H Telenti A. Cd4 T-lymphocyte recovery in individuals with advanced hiv-1 infection receiving potent antiretroviral therapy for 4 years: the swiss hiv cohort study. Arch Internal Med. (2003) 163:2187–95. doi: 10.1001/archinte.163.18.2187 14557216

[4] Kaufmann GR Furrer H Ledergerber B Perrin L Opravil M Vernazza P. Characteristics, determinants, and clinical relevance of cd4 T cell recovery to <500 cells/microl in hiv type 1-infected individuals receiving potent antiretroviral therapy. Clin Infect Dis: An Off Publ Infect Dis Soc America. (2005) 41:361–72. doi: 10.1086/431484 16007534

[5] Gutierrez F Padilla S Masiá M Iribarren JA Moreno S Viciana P. Clinical outcome of hiv-infected patients with sustained virologic response to antiretroviral therapy: long-term follow-up of a multicenter cohort. PloS One. (2006) 1:e89. doi: 10.1371/journal.pone.0000089 17183720 PMC1762396

[6] Kelley CF Kitchen CM Hunt PW Rodriguez B Hecht FM Kitahata M. Incomplete peripheral cd4+ Cell count restoration in hiv-infected patients receiving long-term antiretroviral treatment. Clin Infect Dis: An Off Publ Infect Dis Soc America. (2009) 48:787–94. doi: 10.1086/597093 PMC2720023 19193107

[7] van Lelyveld SF Gras L Kesselring A Zhang S De Wolf F Wensing AM. Long-term complications in patients with poor immunological recovery despite virological successful haart in dutch athena cohort. AIDS (London England). (2012) 26:465–74. doi: 10.1097/QAD.0b013e32834f32f8 22112603

[8] O’Connor JL Smith CJ Lampe FC Hill T Gompels M Hay P. Failure to achieve a cd4+ Cell count response on combination antiretroviral therapy despite consistent viral load suppression. AIDS (London England). (2014) 28:919–24. doi: 10.1097/qad.0000000000000165 24335482

[9] He L Pan X Dou Z Huang P Zhou X Peng Z. The factors related to cd4+ T-cell recovery and viral suppression in patients who have low cd4+ T cell counts at the initiation of haart: A retrospective study of the national hiv treatment sub-database of Zhejiang Province, China, 2014. PloS One. (2016) 11:e0148915. doi: 10.1371/journal.pone.0148915 26900702 PMC4764673

[10] Kroeze S Ondoa P Kityo CM Siwale M Akanmu S Wellington M. Suboptimal immune recovery during antiretroviral therapy with sustained hiv suppression in sub-saharan africa. AIDS (London England). (2018) 32:1043–51. doi: 10.1097/qad.0000000000001801 29547445

[11] Han WM Ubolyam S Apornpong T Kerr SJ Hansasuta P Gatechompol S. Characteristics of Suboptimal Immune Response after Initiating Antiretroviral Therapy among People Living with Hiv with a Pre-Treatment Cd4 T Cell Count <200 ​Cells/Mm(3) in Thailand. J Virus Eradication. (2020) 6:100005. doi: 10.1016/j.jve.2020.100005 PMC7646671 33251023

[12] Neuhaus J Jacobs DR Jr. Baker JV Calmy A Duprez D La Rosa A. Markers of inflammation, coagulation, and renal function are elevated in adults with hiv infection. J Infect Dis. (2010) 201:1788–95. doi: 10.1086/652749 PMC2872049 20446848

[13] Deeks SG. Hiv infection, inflammation, immunosenescence, and aging. Annu Rev Med. (2011) 62:141–55. doi: 10.1146/annurev-med-042909-093756 PMC3759035 21090961

[14] Tenorio AR Zheng Y Bosch RJ Krishnan S Rodriguez B Hunt PW. Soluble markers of inflammation and coagulation but not T-cell activation predict non-aids-defining morbid events during suppressive antiretroviral treatment. J Infect Dis. (2014) 210:1248–59. doi: 10.1093/infdis/jiu254 PMC4192039 24795473

[15] Psomas C Younas M Reynes C Cezar R Portalès P Tuaillon E. One of the immune activation profiles observed in hiv-1-infected adults with suppressed viremia is linked to metabolic syndrome: the activih study. EBioMedicine. (2016) 8:265–76. doi: 10.1016/j.ebiom.2016.05.008 PMC4919610 27428436

[16] Fauci AS Lane HC. Four decades of hiv/aids - much accomplished, much to do. N Engl J Med. (2020) 383:1–4. doi: 10.1056/NEJMp1916753 32609976

[17] Heaton RK Clifford DB Franklin DR Jr. Woods SP Ake C Vaida F. Hiv-associated neurocognitive disorders persist in the era of potent antiretroviral therapy: charter study. Neurology. (2010) 75:2087–96. doi: 10.1212/WNL.0b013e318200d727 PMC2995535 21135382

[18] Heaton RK Franklin DR Ellis RJ McCutchan JA Letendre SL Leblanc S. Hiv-associated neurocognitive disorders before and during the era of combination antiretroviral therapy: differences in rates, nature, and predictors. J Neurovirol. (2011) 17:3–16. doi: 10.1007/s13365-010-0006-1 21174240 PMC3032197

[19] Ulfhammer G Edén A Antinori A Brew BJ Calcagno A Cinque P. Cerebrospinal fluid viral load across the spectrum of untreated human immunodeficiency virus type 1 (Hiv-1) infection: A cross-sectional multicenter study. Clin Infect Dis: An Off Publ Infect Dis Soc America. (2022) 75:493–502. doi: 10.1093/cid/ciab943 PMC9427147 34747481

[20] Thompson PM Dutton RA Hayashi KM Lu A Lee SE Lee JY. 3d mapping of ventricular and corpus callosum abnormalities in hiv/aids. NeuroImage. (2006) 31:12–23. doi: 10.1016/j.neuroimage.2005.11.043 16427319

[21] Adeli E Zahr NM Pfefferbaum A Sullivan EV Pohl KM. Novel machine learning identifies brain patterns distinguishing diagnostic membership of human immunodeficiency virus, alcoholism, and their comorbidity of individuals. Biol Psychiatry Cogn Neurosci Neuroimaging. (2019) 4:589–99. doi: 10.1016/j.bpsc.2019.02.003 PMC6556407 30982583

[22] Pfefferbaum A Zahr NM Sassoon SA Kwon D Pohl KM Sullivan EV. Accelerated and premature aging characterizing regional cortical volume loss in human immunodeficiency virus infection: contributions from alcohol, substance use, and hepatitis C coinfection. Biol Psychiatry Cogn Neurosci Neuroimaging. (2018) 3:844–59. doi: 10.1016/j.bpsc.2018.06.006 PMC6508083 30093343

[23] Tesic T Boban J Bjelan M Todorovic A Kozic D Brkic S. Basal ganglia shrinkage without remarkable hippocampal atrophy in chronic aviremic hiv-positive patients. J Neurovirol. (2018) 24:478–87. doi: 10.1007/s13365-018-0635-3 29687405

[24] O’Connor EE Zeffiro T Lopez OL Becker JT Zeffiro T. Hiv infection and age effects on striatal structure are additive. J Neurovirol. (2019) 25:480–95. doi: 10.1007/s13365-019-00747-w PMC10488234 31028692

[25] Ances BM Hammoud DA. Neuroimaging of hiv-associated neurocognitive disorders (Hand). Curr Opin HIV AIDS. (2014) 9:545–51. doi: 10.1097/coh.0000000000000112 PMC4217490 25250553

[26] Nir TM Fouche JP Ananworanich J Ances BM Boban J Brew BJ. Association of immunosuppression and viral load with subcortical brain volume in an international sample of people living with hiv. JAMA Network Open. (2021) 4:e2031190. doi: 10.1001/jamanetworkopen.2020.31190 33449093 PMC7811179

[27] Yang X Su B Zhang X Liu Y Wu H Zhang T. Incomplete immune reconstitution in hiv/aids patients on antiretroviral therapy: challenges of immunological non-responders. J Leukocyte Biol. (2020) 107:597–612. doi: 10.1002/jlb.4mr1019-189r 31965635 PMC7187275

[28] Dahl V Peterson J Fuchs D Gisslen M Palmer S Price RW. Low Levels of Hiv-1 Rna Detected in the Cerebrospinal Fluid after up to 10 Years of Suppressive Therapy Are Associated with Local Immune Activation. AIDS (London England). (2014) 28:2251–8. doi: 10.1097/qad.0000000000000400 PMC4492794 25022595

[29] Saylor D Dickens AM Sacktor N Haughey N Slusher B Pletnikov M. Hiv-associated neurocognitive disorder–pathogenesis and prospects for treatment. Nat Rev Neurol. (2016) 12:234–48. doi: 10.1038/nrneurol.2016.27 PMC4937456 26965674

[30] Chang L Shukla DK. Imaging studies of the hiv-infected brain. Handb Clin Neurol. (2018) 152:229–64. doi: 10.1016/b978-0-444-63849-6.00018-9 29604980

[31] Fois AF Brew BJ. The potential of the cns as a reservoir for hiv-1 infection: implications for hiv eradication. Curr HIV/AIDS Rep. (2015) 12:299–303. doi: 10.1007/s11904-015-0257-9 25869939

[32] Rojas-Celis V Valiente-Echeverría F Soto-Rifo R Toro-Ascuy D. New challenges of hiv-1 infection: how hiv-1 attacks and resides in the central nervous system. Cells. (2019) 8:1245. doi: 10.3390/cells8101245 31614895 PMC6829584

[33] Sénécal V Barat C Tremblay MJ. The delicate balance between neurotoxicity and neuroprotection in the context of hiv-1 infection. Glia. (2021) 69:255–80. doi: 10.1002/glia.23904 32910482

[34] Rubin LH Langenecker SA Phan KL Keating SM Neigh GN Weber KM. Remitted depression and cognition in hiv: the role of cortisol and inflammation. Psychoneuroendocrinology. (2020) 114:104609. doi: 10.1016/j.psyneuen.2020.104609 32062371 PMC7254879

[35] Hallisey M Dennis J Gabriel EP Masciarelli A Chen J Abrecht C. Profiling of natural killer interactions with cancer cells using mass cytometry. Lab Investigation J Tech Methods Pathol. (2023) 103:100174. doi: 10.1016/j.labinv.2023.100174 37169083

[36] Chang L Holt JL Yakupov R Jiang CS Ernst T. Lower cognitive reserve in the aging human immunodeficiency virus-infected brain. Neurobiol Aging. (2013) 34:1240–53. doi: 10.1016/j.neurobiolaging.2012.10.012 PMC3984923 23158761

[37] Maki PM Cohen MH Weber K Little DM Fornelli D Rubin LH. Impairments in memory and hippocampal function in hiv-positive vs hiv-negative women: A preliminary study. Neurology. (2009) 72:1661–8. doi: 10.1212/WNL.0b013e3181a55f65 PMC2683643 19433739

[38] Chang L Speck O Miller EN Braun J Jovicich J Koch C. Neural correlates of attention and working memory deficits in hiv patients. Neurology. (2001) 57:1001–7. doi: 10.1212/wnl.57.6.1001 11571324

[39] Schweinsburg BC Scott JC Schweinsburg AD Jacobus J Theilmann RJ Frank LR. Altered prefronto-striato-parietal network response to mental rotation in hiv. J Neurovirol. (2012) 18:74–9. doi: 10.1007/s13365-011-0072-z PMC3729929 22271019

[40] Koenigs M Grafman J. The functional neuroanatomy of depression: distinct roles for ventromedial and dorsolateral prefrontal cortex. Behav Brain Res. (2009) 201:239–43. doi: 10.1016/j.bbr.2009.03.004 PMC2680780 19428640

[41] Seghier ML Price CJ. Interpreting and utilising intersubject variability in brain function. Trends Cogn Sci. (2018) 22:517–30. doi: 10.1016/j.tics.2018.03.003 PMC5962820 29609894

[42] Thomas JB Brier MR Snyder AZ Vaida FF Ances BM. Pathways to neurodegeneration: effects of hiv and aging on resting-state functional connectivity. Neurology. (2013) 80:1186–93. doi: 10.1212/WNL.0b013e318288792b PMC3691785 23446675

[43] Ortega M Brier MR Ances BM. Effects of hiv and combination antiretroviral therapy on cortico-striatal functional connectivity. AIDS (London England). (2015) 29:703–12. doi: 10.1097/qad.0000000000000611 PMC4391231 25849834

[44] Ipser JC Brown GG Bischoff-Grethe A Connolly CG Ellis RJ Heaton RK. Hiv infection is associated with attenuated frontostriatal intrinsic connectivity: A preliminary study. J Int Neuropsychol Society: JINS. (2015) 21:203–13. doi: 10.1017/s1355617715000156 PMC4400233 25824201

[45] Melrose RJ Tinaz S Castelo JM Courtney MG Stern CE. Compromised fronto-striatal functioning in hiv: an fmri investigation of semantic event sequencing. Behav Brain Res. (2008) 188:337–47. doi: 10.1016/j.bbr.2007.11.021 18242723

[46] Ettenhofer ML Foley J Castellon SA Hinkin CH. Reciprocal prediction of medication adherence and neurocognition in hiv/aids. Neurology. (2010) 74:1217–22. doi: 10.1212/WNL.0b013e3181d8c1ca PMC2865732 20220123

[47] Chang L Ernst T Leonido-Yee M Witt M Speck O Walot I. Highly active antiretroviral therapy reverses brain metabolite abnormalities in mild hiv dementia. Neurology. (1999) 53:782–9. doi: 10.1212/wnl.53.4.782 10489041

[48] Gisslén M Krut J Andreasson U Blennow K Cinque P Brew BJ. Amyloid and tau cerebrospinal fluid biomarkers in hiv infection. BMC Neurol. (2009) 9:63. doi: 10.1186/1471-2377-9-63 20028512 PMC2807422

[49] Zhang Y Ji J Xie K Cai M Wang R Zhang X. Pathological proliferation: A potential mechanism for poor cd4+ T cell recovery in people living with hiv. Front Cell Infect Microbiol. (2024) 14:1344778. doi: 10.3389/fcimb.2024.1344778 38601742 PMC11004319

[50] Booiman T Wit FW Maurer I De Francesco D Sabin CA Harskamp AM. High cellular monocyte activation in people living with human immunodeficiency virus on combination antiretroviral therapy and lifestyle-matched controls is associated with greater inflammation in cerebrospinal fluid. Open Forum Infect Dis. (2017) 4:ofx108. doi: 10.1093/ofid/ofx108 28680905 PMC5494939

[51] Huaman MA Juchnowski SM Zidar DA Kityo CM Nalukwago S Nazzinda R. Monocyte activation in persons living with hiv and tuberculosis coinfection. AIDS (London England). (2021) 35:447–52. doi: 10.1097/qad.0000000000002766 PMC7855758 33252496

[52] Wilms H Zecca L Rosenstiel P Sievers J Deuschl G Lucius R. Inflammation in parkinson’s diseases and other neurodegenerative diseases: cause and therapeutic implications. Curr Pharm Design. (2007) 13:1925–8. doi: 10.2174/138161207780858429 17584117

[53] Conant K Garzino-Demo A Nath A McArthur JC Halliday W Power C. Induction of monocyte chemoattractant protein-1 in hiv-1 tat-stimulated astrocytes and elevation in aids dementia. Proc Natl Acad Sci United States America. (1998) 95:3117–21. doi: 10.1073/pnas.95.6.3117 PMC19704 9501225

[54] Lelièvre JD Petit F Arnoult D Ameisen JC Estaquier J. Interleukin 7 increases human immunodeficiency virus type 1 lai-mediated fas-induced T-cell death. J Virol. (2005) 79:3195–9. doi: 10.1128/jvi.79.5.3195-3199.2005 PMC548424 15709041

[55] Tang WW Ulich TR Lacey DL Hill DC Qi M Kaufman SA. Platelet-derived growth factor-bb induces renal tubulointerstitial myofibroblast formation and tubulointerstitial fibrosis. Am J Pathol. (1996) 148:1169–80. PMC1861538 8644858

[56] Salahuddin MF Mahdi F Paris JJ. Hiv-1 tat dysregulates the hypothalamic-pituitary-adrenal stress axis and potentiates oxycodone-mediated psychomotor and anxiety-like behavior of male mice. Int J Mol Sci. (2020) 21:8212. doi: 10.3390/ijms21218212 33153023 PMC7662349

