# Supplementary material for: Unveiling neuroimmunology profile of immunological non-responders in HIV: a multimodal MRI approach
Source: Front Immunol. 2024 Dec 13;15:1452532. doi: 10.3389/fimmu.2024.1452532 (PMC11671397; doi:10.3389/fimmu.2024.1452532)
Supplement: Supplementary file 1 [file Table1.docx]

Supplementary Material

**Supplementary Table 1 Antibodies and isotopes used for mass cytometry**

|  | **Antibodies Name** | **Isotopes Name** | **Clone** |
| --- | --- | --- | --- |
| 1 | Anti-Human CD16 | 209Bi | 3GB |
| 2 | Anti-Human CD57 | 142Nd | HCD57 |
| 3 | Anti-Human KI67 | 161Dy | B56 |
| 4 | Anti-Human FOXP3 | 162Dy | PCH101 |
| 5 | Anti-Human CCR7 | 167Er | G403H7 |
| 6 | Anti-Human CD127 | 168Er | A019D5 |
| 7 | Anti-Human HLA-DR | 170Er | L243 |
| 8 | Anti-Human CD107A | 151Eu | H4A3 |
| 9 | Anti-Human CCR2 | 153Eu | K036C2 |
| 10 | Anti-Human CD163 | 154Sm | GHI/61 |
| 11 | Anti-Human PD-1 | 155Gd | EH12.2H7 |
| 12 | Anti-Human PD-L1 | 156Gd | 29E.2A3 |
| 13 | Anti-Human PERFORIN | 175Lu | B-D48 |
| 14 | Anti-Human CD45RA | 143Nd | Hl100 |
| 15 | Anti-Human CD4 | 145Nd | RPA-T4 |
| 16 | Anti-Human CD8 | 146Nd | RPA-T8 |
| 17 | Anti-Human CD3 | 141Pr | UCHT1 |
| 18 | Anti-Human CD31 | 144Nd | WM59 |
| 19 | Anti-Human CD14 | 148Nd | RM052 |
| 20 | Anti-Human CD25 | 149Sm | 2A3 |
| 21 | Anti-Human CD86 | 150Nd | IT2.2 |
| 22 | Anti-Human CD45 | 89Y | HI30 |
| 23 | Anti-Human CD38 | 172Yb | HIT2 |

**Supplementary Table 2 T cell and monocyte clusters of all participants**

|  | **Subpopulation** | **Clusters** | **Markers** |
| --- | --- | --- | --- |
| CD4^+^ T cells | Naïve T cells | C20 | CD3^+^ CD4^+^ CD8^-^ CD45RA^+^ CCR7^+^ |
|  | Central memory T cells | C7, C8, C9, C10, C12 | CD3^+^ CD4^+^ CD8^-^ CD45RA^-^ CCR7^+^ |
|  | Effector memory T cells | C6, C17 | CD3^+^ CD4^+^ CD8^-^ CD45RA^-^ CCR7^-^ |
| CD8^+^ T cells | Naïve T cells | C4, C11, C14, C16 | CD3^+^ CD4^-^ CD8^+^ CD45RA^+^ CCR7^+^ |
|  | CD45RA^+^ effector memory T cells | C2, C23 | CD3^+^ CD4^-^ CD8^+^ CD45RA^+^ CCR7^-^ |
|  | Central memory T cells | C15, C21, C22 | CD3^+^ CD4^-^ CD8^+^ CD45RA^-^ CCR7^+^ |
|  | Effector memory T cells | C1, C3, C24 | CD3^+^ CD4^-^ CD8^+^ CD45RA^-^ CCR7^-^ |
| CD4^+^ CD8^+^ T cells |  | C13, C18, C25 | CD3^+^ CD4^+^ CD8^+^ |
| CD4^-^ CD8^-^ T cells |  | C9, C5 | CD3^+^ CD4^-^ CD8^-^ |
| Monocytes | Classical monocytes | C4, C7, C8, C11, C15, C16, C19 | CD3^-^ CD14^+^ CD16^-^ |
|  | Intermediate monocytes | C1, C2, C3, C5, C9, C13, C14, C17, C18 | CD3^-^ CD14^+^ CD16^+^ |
|  | Non-classical monocytes | C6, C10, C12 | CD3^-^ CD14^+^ CD16^++^ |

Supplementary Table 3 ALFF, ReHo, and GMV Alterations in Brain Regions Between INRs and IRs

| Brain areas | MNI coordinates (X, Y, Z) mm | Number of voxels | Peak t-value | Cluster-level P value | Voxel-level P value | Defination of ROI |
| --- | --- | --- | --- | --- | --- | --- |
| Lingual_L | -19.5, -43.5, -1.5 | 20 | 3.3185 | 0.731 | 0.001 | 1 |
| Rolandic_Oper_R | 60, 10.5, 6 | 56 | 3.8032 | 0.536 | 0.000 | 2 |
| Occipital_Mid_L | -37.5, -76.5, 10.5 | 36 | 3.5326 | 0.629 | 0.000 | 3 |
| Temporal_Sup_L | -63, -54, 19.5 | 25 | 3.6032 | 0.695 | 0.000 | 4 |
| Postcentral_L | -51, -15, 40.5 | 14 | 3.3615 | 0.781 | 0.001 | 5 |
| Temporal_Mid_L | -51, -18, -21 | 17 | 5.1044 | 0.010 | 0.000 | 6 |
| Frontal_Inf_Tri_R | 57, 39, 0 | 13 | 4.0634 | 0.020 | 0.000 | 7 |
| Frontal_Sup_Medial_R | 9, 66, 9 | 20 | 4.3333 | 0.006 | 0.000 | 8 |
| Frontal_Inf_Tri_L | -51, 24, 18 | 10 | 3.6742 | 0.038 | 0.000 | 9 |
| Frontal_Inf_Tri_L | -51, 33, 0 | 10 | 4.3351 | 0.006 | 0.000 | 9 |
| Thalamus_L | -15, -21, 6 | 12 | 4.3299 | 0.003 | 0.000 | 10 |
| Temporal_Mid_L | -48, -18, -21 | 34 | 4.7472 | 0.017 | 0.000 | 6 |

Notes: This table presents significant alterations in brain regions between immunological non-responders (INRs) and immunological responders (IRs), analyzed for Amplitude of Low-Frequency Fluctuations (ALFF), Regional Homogeneity (ReHo), and Gray Matter Volume (GMV). The regions are identified by their Montreal Neurological Institute (MNI) coordinates (X, Y, Z, in millimeters), number of voxels in the cluster, peak t-value, cluster-level p-value, voxel-level p-value, and the Region of Interest (ROI) definition. Statistical significance was determined using a voxel-level p-value threshold of < 0.001 or a cluster-level threshold of < 0.05. Abbreviations: ALFF, Amplitude of Low-Frequency Fluctuations; ReHo, Regional Homogeneity; GMV, Gray Matter Volume; MNI, Montreal Neurological Institute; ROI, Region of Interest; Lingual_L: Left Lingual Gyrus; Rolandic_Oper_R: Right Rolandic Operculum; Occipital_Mid_L: Left Middle Occipital Gyrus; Temporal_Sup_L: Left Superior Temporal Gyrus; Postcentral_L: Left Postcentral Gyrus; Temporal_Mid_L: Left Middle Temporal Gyrus; Frontal_Inf_Tri_R: Right Inferior Frontal Gyrus, Triangular Part; Frontal_Sup_Medial_R: Right Superior Medial Frontal Gyrus; Frontal_Inf_Tri_L: Left Inferior Frontal Gyrus, Triangular Part; Thalamus_L: Left Thalamus.

Supplementary Table 4 Voxel-wise FC Alterations Between Right Superior Medial Frontal Gyrus and Global Brain Regions

| FC alteration | Connected areas | MNI coordinates (X, Y, Z) mm | Number of voxels | Peak t-value | Cluster-level *P* value | Voxel-level *P* value |
| --- | --- | --- | --- | --- | --- | --- |
| Decreased | Frontal_Med_Orb_L | 0, 66, -9 | 29 | 4.9843 | 0.000 | 0.000 |
|  | Frontal_Sup_Medial_R | 3, 63, 24 | 610 | 6.1806 | 0.000 | 0.000 |
|  | Precuneus_L | -9, -54, 21 | 37 | 4.3262 | 0.000 | 0.000 |
|  | Angular_R | 51, -66, 36 | 15 | 3.9956 | 0.000 | 0.000 |
|  | Angular_L | -54, -63, 30 | 16 | 5.0127 | 0.000 | 0.000 |
|  | Precuneus_R | 3, -63, 45 | 21 | 4.0904 | 0.000 | 0.000 |
|  | Frontal_Mid_L | -30, 30, 54 | 13 | 4.7898 | 0.006 | 0.000 |
| Increased | Rolandic_Oper_R | 60, 9, 0 | 574 | 7.4293 | 0.000 | 0.000 |
|  | Rolandic_Oper_L | -42, -12, 15 | 265 | 6.1283 | 0.000 | 0.000 |

Notes: This table shows voxel-wise functional connectivity (FC) alterations between the right superior medial frontal gyrus (Frontal_Sup_Medial_R) and other brain regions. FC alterations are categorized as either decreased or increased, with connected brain regions identified alongside their Montreal Neurological Institute (MNI) coordinates (X, Y, Z, in millimeters), the number of voxels in the cluster, peak t-value, and cluster-level p-value. Statistical significance was determined using a voxel-level p-value threshold of < 0.001 or a cluster-level threshold of < 0.05. FC1–FC9:The FC measures represent the connectivity of the Right Superior Medial Frontal Gyrus (ROI8) with the following brain regions: FC1: Frontal_Med_Orb_L; FC2: Frontal_Sup_Medial_R; FC3: Precuneus_L; FC4: Angular_R; FC5: Angular_L; FC6: Precuneus_R; FC7: Frontal_Mid_L; FC8: Rolandic_Oper_R; FC9: Rolandic_Oper_L. Abbreviations: FC, Functional Connectivity; MNI, Montreal Neurological Institute; ROI, Region of Interest; Frontal_Med_Orb_L: Left Medial Orbital Frontal Gyrus; Frontal_Sup_Medial_R: Right Superior Medial Frontal Gyrus; Precuneus_L: Left Precuneus; Angular_R: Right Angular Gyrus; Angular_L: Left Angular Gyrus; Precuneus_R: Right Precuneus; Frontal_Mid_L: Left Middle Frontal Gyrus; Rolandic_Oper_R: Right Rolandic Operculum； Rolandic_Oper_L: Left Rolandic Operculum.

Supplementary Table 5 Correlations Between Brain Functional Alterations and Peripheral Immune Markers

| Neuroimaging | Peripheral Immune Markers | | Spearman’S R | | *P* Value |
| --- | --- | --- | --- | --- | --- |
| MTG.L ALFF | VEGF-A | -0.443 | | < 0.05 | |
| IFGtriang.R ALFF | IGF-1 | -0.451 | | < 0.05 | |
| IFGtriang.L ALFF | IL-12(p40) | -0.402 | | < 0.05 | |
|  | MIP-1alpha | -0.413 | | < 0.05 | |
| IFGtriang.L fALFF | CORT | -0.399 | | < 0.05 | |
|  | IL-10 | 0.411 | | < 0.05 | |
|  | IL-3 | 0.406 | | < 0.05 | |
| THA.L ReHo | Eotaxin | -0.464 | | < 0.05 | |
| MTG.L.ReHo | BNGF | 0.517 | | < 0.01 | |
|  | IL-5 | -0.403 | | < 0.05 | |
| FC2 | Eotaxin | -0.413 | | < 0.05 | |
|  | IGF-1 | 0.469 | | < 0.05 | |
| FC3 | IL-10 | 0.491 | | < 0.05 | |
|  | IL-12(p40) | 0.517 | | < 0.01 | |
|  | IL-17F | 0.422 | | < 0.05 | |
|  | IL-18 | 0.434 | | < 0.05 | |
|  | IL-1RA | 0.527 | | < 0.01 | |
|  | IL-7 | 0.447 | | < 0.05 | |
|  | IL-9 | 0.445 | | < 0.05 | |
|  | TGF-alpha | 0.437 | | < 0.05 | |
|  | TNF-alpha | 0.514 | | < 0.01 | |
| FC4 | IL-18 | 0.411 | | < 0.05 | |
| FC5 | BNGF | 0.477 | | < 0.05 | |
|  | CORT | 0.4 | | < 0.05 | |
|  | IL-12(p40) | 0.441 | | < 0.05 | |
|  | IL-17F | 0.42 | | < 0.05 | |
|  | IL-18 | 0.464 | | < 0.05 | |
|  | IL-1RA | 0.427 | | < 0.05 | |
|  | IL-1alpha | 0.432 | | < 0.05 | |
|  | CD57 of C15 in T cells | 0.474 | | < 0.05 | |
|  | CD57 of C19 in T cells | 0.411 | | < 0.05 | |
| FC6 | IL-7 | 0.584 | | < 0.01 | |
|  | IP-10 | 0.685 | | < 0.001 | |
|  | MIG | 0.464 | | < 0.05 | |
|  | PDGF-AA | 0.405 | | < 0.05 | |
|  | PDGF-AB/BB | 0.526 | | < 0.01 | |
|  | TNF-alpha | 0.407 | | < 0.05 | |
|  | sCD40L | 0.445 | | < 0.05 | |
|  | CD57 of C10 in T cells | 0.415 | | < 0.05 | |
|  | CD57 of C19 in T cells | 0.609 | | < 0.01 | |
|  | CD57 of C20 in T cells | 0.481 | | < 0.05 | |
| FC7 | CD57 of C20 in T cells | -0.428 | | < 0.05 | |
| FC8 | G-CSF | 0.453 | | < 0.05 | |
|  | IL-12(p40) | 0.481 | | < 0.05 | |
|  | IL-18 | 0.475 | | < 0.05 | |
|  | IL-1RA | 0.497 | | < 0.05 | |
|  | IL-7 | 0.399 | | < 0.05 | |
|  | IL-9 | 0.476 | | < 0.05 | |
|  | IP-10 | 0.454 | | < 0.05 | |
|  | TNF-alpha | 0.424 | | < 0.05 | |
|  | sCD40L | 0.417 | | < 0.05 | |
| FC9 | BDNF | -0.423 | | < 0.05 | |

Notes : This table presents the Spearman correlation analysis results between brain functional alterations and peripheral immune markers. Neuroimaging measures, including Amplitude of Low-Frequency Fluctuations (ALFF), Fractional ALFF (fALFF), Regional Homogeneity (ReHo), and Functional Connectivity (FC), were analyzed in relation to immune markers such as cytokines, chemokines, and T cell properties. Each row includes the brain region or FC measure, the correlated immune marker, the Spearman’s correlation coefficient (R), and the p-value for significance. Only correlations with p-values < 0.05 are reported. FC1–FC9:The FC measures represent the connectivity of the Right Superior Medial Frontal Gyrus (ROI8) with the following brain regions: FC1: Frontal_Med_Orb_L; FC2: Frontal_Sup_Medial_R; FC3: Precuneus_L; FC4: Angular_R; FC5: Angular_L; FC6: Precuneus_R; FC7: Frontal_Mid_L; FC8: Rolandic_Oper_R; FC9: Rolandic_Oper_L. Abbreviations: MTG.L: Left Middle Temporal Gyrus; IFGtriang.R: Right Inferior Frontal Gyrus, Triangular Part; IFGtriang.L: Left Inferior Frontal Gyrus, Triangular Part; THA.L: Left Thalamus; BNGF: Brain-Derived Neurotrophic Factor; MTG.L: Left Middle Temporal Gyrus; IFGtriang.R: Right Inferior Frontal Gyrus, Triangular Part; IFGtriang.L: Left Inferior Frontal Gyrus, Triangular Part; THA.L: Left Thalamus; BNGF: Brain-Derived Neurotrophic Factor.
